# 1361. The association between antibody responses and prolonged viable SARS-CoV-2 shedding in immunocompromised patients: a prospective cohort study

**DOI:** 10.1093/ofid/ofad500.1198

**Published:** 2023-11-27

**Authors:** So Yun Lim, Jun-Won Kim, Ji Yeun Kim, Sung-Woon Kang, Choi-Young Jang, Eui Jin Chang, Sung-Cheol Yun, Jeong-Sun Yang, Kyung-Chang Kim, Hee-Chang Jang, Dasol Kim, Younmin Shin, Joo-Yeon Lee, Sung-Han Kim

**Affiliations:** National Medical Center , Seoul, Seoul-t'ukpyolsi, Republic of Korea; National Institute of Infectious Diseases, Cheong-ju, Ch'ungch'ong-bukto, Republic of Korea; Asan Medical Center, Seoul, Seoul-t'ukpyolsi, Republic of Korea; Asan Medical Center, Seoul, Seoul-t'ukpyolsi, Republic of Korea; Asan Medical Center, Seoul, Seoul-t'ukpyolsi, Republic of Korea; Department of Internal Medicine, Asan Medical Center, Seoul, Korea, Seoul, Seoul-t'ukpyolsi, Republic of Korea; Asan medical center, Seoul, Seoul-t'ukpyolsi, Republic of Korea; National Institute of Infectious Diseases, Cheong-ju, Ch'ungch'ong-bukto, Republic of Korea; National Institute of Infectious Diseases, Cheong-ju, Ch'ungch'ong-bukto, Republic of Korea; National Institute of Infectious Diseases, Cheong-ju, Ch'ungch'ong-bukto, Republic of Korea; National Institute of Infectious Diseases, Cheong-ju, Ch'ungch'ong-bukto, Republic of Korea; National Institute of Infectious Diseases, Cheong-ju, Ch'ungch'ong-bukto, Republic of Korea; National Institute of Infectious Diseases, Cheong-ju, Ch'ungch'ong-bukto, Republic of Korea; Asan medical center, Seoul, Seoul-t'ukpyolsi, Republic of Korea

## Abstract

**Background:**

Immunocompromised patients have been shown to have prolonged SARS-CoV-2 viral shedding. However, there are limited data on the longitudinal association between immune response and viable virus shedding in immunocompromised patients. Thus, we aimed to investigate the relationship between the kinetics of the immune responses and the duration of viable virus shedding in immunocompromised patients.

**Methods:**

We prospectively enrolled immunocompromised patients with COVID-19 who were admitted to a tertiary center in Seoul, South Korea, from March 2022 to August 2022. SARS-CoV-2 S1-specific IgG antibody and neutralizing antibody were measured by ELISA and plaque reduction neutralizing assay, respectively. Genomic RNA, subgenomic RNA, and culture-based virus isolation were performed on respiratory samples to identify viral shedding.

**Results:**

A total of 62 patients whose serial blood and respiratory samples were obtained were analyzed. Significant group (degree of antibody response)-by-time (interval from the infection) interaction was observed in terms of both S1-IgG antibody (P < 0.001) and neutralizing antibody (P < 0.001), that is, the genomic RNA declined significantly more rapidly in patients with higher antibody response compared with lower antibody response. There was a significant difference in the proportion of culturable virus according to the time from SARS-CoV-2 infection depending on neutralizing antibody level (P=0.04), while there was no difference depending on the S1-IgG antibody level (P=0.06).

**Figure 1.**
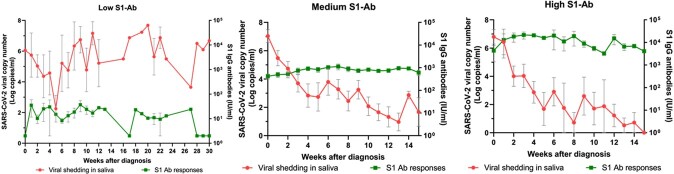
SARS-CoV-2 viral shedding and the degree of S1-specific IgG antibody according to the time from the diagnosis

**Figure 2.**
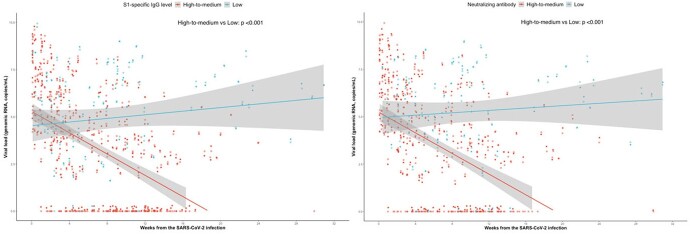
Genomic viral RNA shedding by time from the SARS-CoV-2 infection depending on the degree of antibody responses. Left panel. S1-IgG antibody Right panel. Neutralizing antibody

**Figure 3.**
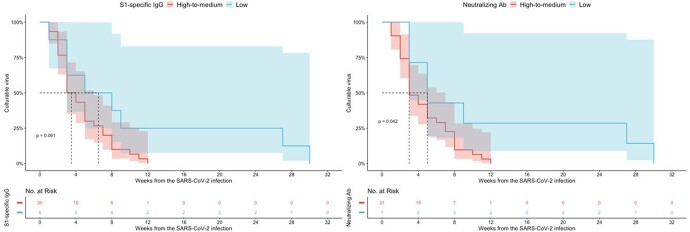
Proportion of culturable virus shedding by time from the SARS-CoV-2 infection depending on the degree of antibody responses.

**Conclusion:**

Our findings suggest that neutralizing antibody response is an important factor associated with prolonged viable SARS-CoV-2 shedding in immunocompromised patients with COVID-19. These findings provide us important insight into the pathophysiology of viral clearance and the potential role of boosting humoral immune response against COVID-19 in immunocompromised patients including vaccination and monoclonal antibody prophylaxis or therapy.

**Table 1.**
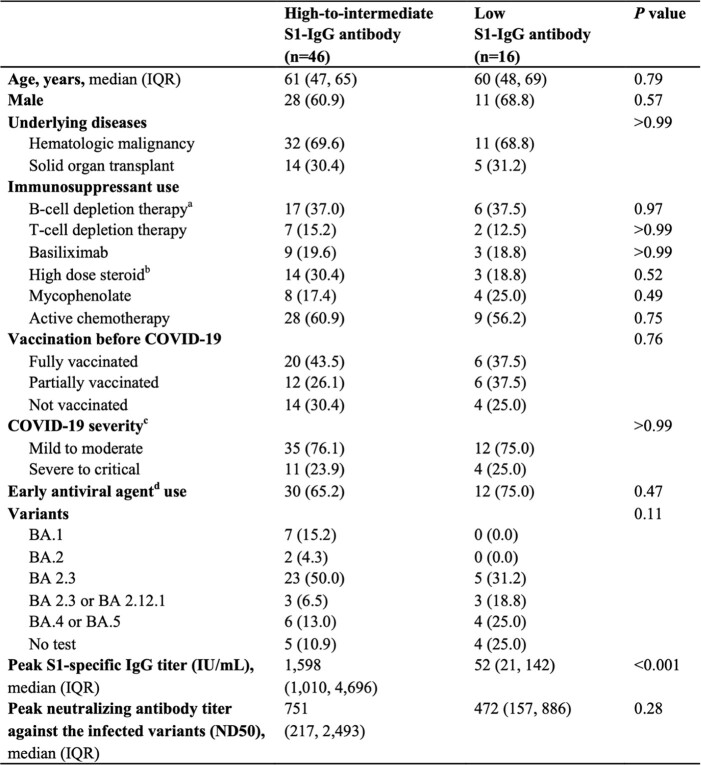
Baseline characteristics of the study participants. Data represents n(%) unless otherwise indicated aRituximab or bispecific T-cell engager b≥0.3 mg/kg corticosteroids for ≥3 weeks in the past 60 days cNational Institue of Health (NIH) COVID-19 severity classification dRemdesivir or nirmatrelvir/ritonavir within 5 days of diagnosis

**Disclosures:**

**All Authors**: No reported disclosures

